# Detection of a Highly Divergent Type 3 Vaccine-Derived Poliovirus in a Child with a Severe Primary Immunodeficiency Disorder — Chongqing, China, 2022

**DOI:** 10.15585/mmwr.mm7136a2

**Published:** 2022-09-09

**Authors:** Ning Yao, Yang Liu, Jia-Wei Xu, Qing Wang, Zun-Dong Yin, Ning Wen, Hong Yang, Lance E. Rodewald, Zhi-Yong Zhang

**Affiliations:** ^1^Chongqing Center for Disease Control and Prevention, Chongqing, China; ^2^Department of Health Statistics, Army Medical University, Chongqing, China; ^3^Chinese Center for Disease Control and Prevention, Beijing, China; ^4^Department of Rheumatology and Immunology, Children’s Hospital of Chongqing Medical University, Chongqing, China; ^5^National Clinical Research Center for Child Health and Disorders, Children’s Hospital of Chongqing Medical University, Chongqing, China; ^6^Ministry of Education Key Laboratory of Child Development and Disorders, Children's Hospital of Chongqing Medical University, Chongqing, China; ^7^Chongqing Key Laboratory of Child Infection and Immunity, Children's Hospital of Chongqing Medical University, Chongqing, China.

Oral poliovirus vaccine (OPV) has proven to be highly effective in the global effort to eradicate poliomyelitis because of its ability to induce both humoral and intestinal immunity, ease of administration, and low cost (*1*). Sabin-strain OPV contains live attenuated virus and induces immunity by replicating in the intestinal tract, triggering an immune response that clears the vaccine virus. However, among undervaccinated communities and persons with immunodeficiency, OPV mutations that arise during prolonged replication can result in the emergence of genetically divergent, neurovirulent vaccine-derived polioviruses (VDPVs). In addition, OPV has resulted in rare cases of vaccine-associated paralytic poliomyelitis (VAPP) among vaccine recipients or their close contacts (*1*). Identification of circulating polioviruses relies on surveillance of acute flaccid paralysis (AFP) and environmental surveillance of wastewater (i.e., sewage). In 2022, type 3 VDPV (VDPV3) was detected in stool specimens from an infant with primary immunodeficiency disorder (PID) through a pilot surveillance program to identify VDPVs in children with PIDs. Integrated AFP, environmental, and immunodeficiency-associated VDPV (iVDPV) surveillance is critical to detecting and containing all polioviruses and achieving the goal of global polio eradication.

In 2016, the year after the Global Polio Eradication Initiative (GPEI) Global Certification Commission* certified the eradication of type 2 wild poliovirus (WPV2) (*2*), China joined a global, synchronized effort to cease the use of type 2 oral poliovirus vaccine (OPV2). At that time, the routine polio vaccination schedule was changed from 3 doses of trivalent OPV (which contains Sabin strain types 1, 2 and 3) to 1 dose of injectable inactivated polio vaccine (IPV) (which contains inactivated 1, 2, and 3 poliovirus serotypes) followed by 3 doses of bivalent OPV (bOPV) (which contains Sabin strain types 1 and 3). In 2020, the schedule was changed to 2 doses of IPV followed by 2 doses of bOPV to increase protection against type 2 poliovirus.

Although China was declared free of all indigenous wild poliovirus (WPV) transmission by the GPEI’s Regional Certification Commission in 2000,^†^ the country continues to face two substantial threats to its polio-free status, namely the risk for importation of WPV from a country with endemic transmission and the emergence of circulating VDPVs because of ongoing domestic use of OPV. Sensitive, nationwide AFP surveillance^§^ is effective in detecting children paralyzed by WPVs and VDPVs. The National Polio Laboratory Network of China supports environmental surveillance to detect polioviruses excreted from infected persons or circulating in a community, even if not detected by AFP surveillance.

Prolonged excretion of iVDPVs can potentially seed community transmission of genetically divergent infectious polioviruses, threatening polio eradication efforts. Children with PIDs are susceptible to recurrent, severe enterovirus infections. Because their immune systems cannot clear replicating live vaccine virus, these children are at increased risk for paralysis when exposed to OPV (*3*). Sensitive AFP surveillance detects iVDPV–infected persons with paralysis; however, persons who excrete iVDPV might not develop paralysis in the short-term, (*4*) and wastewater analysis in China is geographically limited in scope because not all areas of the country conduct environmental surveillance. These limitations of poliovirus surveillance mean that a substantial number of iVDPVs cases might not be routinely detected.

In response to World Health Organization (WHO) recommendations to extend poliovirus surveillance to persons with PIDs, the Chinese Center for Disease Control and Prevention (CCDC) launched a pilot iVDPV surveillance program in 2021. Five children’s hospitals located in Beijing, Shanghai, Zhengzhou, and Chongqing participate in the program, which recruits children who receive a new diagnosis of primary antibody deficiency or combined immunodeficiency disorder to provide stool specimens for poliovirus testing.

In March 2022, VDPV3 was detected in stool specimens from an infant who had received a new diagnosis of PID and was hospitalized in Children’s Hospital of Chongqing Medical University (CHCMU). CCDC and partners in Chongqing investigated the case. This study was reviewed and approved by the institutional review board of the Chinese Center for Disease Control and Prevention.

The patient, a boy aged 1 year, was born in Guizhou province. He was initially admitted to CHCMU’s immunology division at age 6 months with persistent diarrhea, daily fevers, diffuse red papular rash, and lymphadenitis. He received a diagnosis of severe combined immunodeficiency (SCID) with heterozygous mutations in the ZAP70 gene, which is a rare autosomal recessive form of SCID caused by abnormal T-cell receptor signaling. Lymph node biopsy and culture found disseminated mycobacterial disease. He had received the recommended Bacille Calmette-Guérin vaccine (BCG) on the first day of life and subsequently developed recurrent localized abscesses and ulcers at the BCG vaccination site. His parents reported having sought treatment at a local hospital at this time; however, no documentation of any evaluation was available. The patient had also received 2 IPV doses at ages 2 and 3 months (May 24 and June 29, 2021, respectively) and the first bOPV dose at age 4 months (July 29, 2021), as recommended. Shortly after receipt of the first bOPV dose, he experienced left axillary lymphadenitis that ultimately involved right axillary, occipital, and cervical lymph nodes. He later acquired *Klebsiella pneumoniae* and developed *Pneumocystis yersini* pneumonia. He died of respiratory failure in the CHCMU intensive care unit on May 3, 2022, at age 13 months.

During the patient’s hospitalization, stool specimens were obtained on February 28 and March 1, 2022, and sent to the CCDC polio laboratory for testing in accordance with WHO recommendations (*5*). Four isolates obtained and tested by real-time reverse transcription–polymerase chain reaction were identified as type 3 poliovirus. Genetic sequencing of viral capsid VP1 coding region indicated that the four isolates diverged from type 3 Sabin strain by 22, 23, 22, and 24 nucleotides (2.4%–2.7%) and shared 15 nucleotide substitutions.

## Discussion

The first identified iVDPV case was reported in the United Kingdom in 1962; as of May 2020, only 149 cases have been reported worldwide (*6*). Most patients with iVDPV develop paralysis before they receive a diagnosis of immune deficiency and are typically detected through AFP surveillance. Other iVDPV cases have been detected through stool cultures obtained to diagnose enterovirus infection in children with suspected or confirmed PID. Among the three types of poliovirus, 56% of iVDPVs were type 2, 23% were type 3, 17% were type 1, and 4% were heterotypic mixtures (*6*). The incidence of iVDPV2 detection declined markedly after the global removal of OPV2 from routine immunization in 2016. Eleven cases of iVDPV were detected in China by AFP surveillance through 2021, before the case described in this report; among these previous cases, four patients died and seven stopped excreting poliovirus.

Children with PID are affected by a range of inherited disorders that result in developmental defects or dysfunction of immune system components (*7*). Live vaccines are usually contraindicated in children with PID because of their risk for causing disease. Although prenatal screening programs can identify some PIDs, identification and diagnosis of PID requires consultation with specialists including clinical immunologists. Infants with PID might therefore receive BCG or OPV before receiving a diagnosis of PID, increasing the risk for disseminated mycobacterial disease and iVDPV infection. ZAP70 gene deficiency is very rare and manifests with typical clinical features of SCID early in life (*8*). Approximately one half of BCG-vaccinated SCID patients have developed BCG-associated manifestations (*9*). Therefore, dissemination after BCG vaccination might be the initial clinical sign of PID, after which, receipt of live, attenuated vaccines is contraindicated.

As the global initiative progresses toward polio eradication, identification of patients with PID is increasing in importance, because iVDPVs can jeopardize polio eradication efforts through long-term excretion by PID patients. To identify nonparalyzed iVDPV cases, GPEI has proposed augmenting AFP and environmental surveillance with poliovirus surveillance in children with PID diagnoses and is supporting implementation of iVDPV surveillance in several countries (*6*).

The findings in this report are subject to at least one limitation. The infant’s death precluded collection of additional stool specimens to further assess virus mutations. The infant described in this report never experienced paralysis. Among known patients who excrete iVDPVs, approximately 30% do not experience paralysis (*4*).

Surveillance among patients with PID has increased detection of iVDPVs in patients without paralysis (*6*). This early finding of a nonparalyzed iVDPV patient in the PID pilot project supports the development of a long-term plan and guidance for iVDPV surveillance in China. Comprehensive iVDPV surveillance requires awareness among clinical immunologists that children who receive a new diagnosis of PID should have stool specimens tested for poliovirus by contacting their local public health authorities. Currently, antiviral treatment of iVDPV infections is under development (*10*). Effective treatment clears prolonged or chronic infection among patients with PIDs and removes a potential source of poliovirus transmission. Integrated systematic poliovirus surveillance including AFP, environmental, and iVDPV surveillance is critical to detecting and containing all polioviruses and helping to achieve and sustain a world free of polio.

**Summary**

**What is already known about this topic?**

Surveillance of acute flaccid paralysis (AFP) and wastewater (environmental) are critical to polio eradication efforts. Children with primary immunodeficiency disorders (PIDs) can excrete vaccine-derived polioviruses (VDPVs), which can hamper eradication efforts.

**What is added by this report?**

In March 2022, a type 3 VDPV was detected in stool specimens from an infant with PID who was hospitalized in Children’s Hospital of Chongqing Medical University, China. Surveillance for poliovirus in PID patients has increased detection of immunodeficiency-related (iVDPV) cases.

**What are the implications for public health practice?**

Integrated systematic poliovirus surveillance, including AFP, environmental, and iVDPV surveillance, is critical to the detection and containment of all polioviruses and achievement of global polio eradication.

## Footnotes

*
https://polioeradication.org/tools-and-library/policy-reports/certification-reports/global-certification-commission/

†
https://www.who.int/china/health-topics/poliomyelitis-(polio)#:~:text=In%202000%20China%20was%20declared,the%20WHO%20Western%20Pacific%20Region

§ AFP surveillance comprises identification and reporting of children with AFP and transporting of stool specimens to a certified laboratory for analysis. Isolated polioviruses are further characterized to determine a strain’s origin. https://polioeradication.org/polio-today/polio-now/surveillance-indicators/
